# Necessary Conditions for Nonlinear Ultrasonic Modulation Generation Given a Localized Fatigue Crack in a Plate-Like Structure

**DOI:** 10.3390/ma10030248

**Published:** 2017-02-28

**Authors:** Hyung Jin Lim, Hoon Sohn

**Affiliations:** Department of Civil and Environmental Engineering, KAIST, Daejeon 34141, Korea; limnice87@kaist.ac.kr

**Keywords:** nonlinear ultrasonic modulation, fatigue crack detection, necessary conditions, localized nonlinearity, propagating waves, stationary vibrations

## Abstract

It has been shown that nonlinear ultrasonics can be more sensitive to local incipient defects, such as a fatigue crack, than conventional linear ultrasonics. Therefore, there is an increasing interest in utilizing nonlinear ultrasonics for structural health monitoring and nondestructive testing applications. While the conditions, which are the necessary conditions that should be satisfied for the generation of nonlinear harmonic components, are extensively studied for distributed material nonlinearity, little work has been done to understand the necessary conditions at the presence of a localized nonlinear source such as a fatigue crack. In this paper, the necessary conditions of nonlinear ultrasonic modulation generation in a plate-like structure are formulated specifically for a localized nonlinear source. Then, the correctness of the formulated necessary conditions is experimentally verified using ultrasounds obtained from aluminum plates.

## 1. Introduction

Due to a high sensitivity to micro defects such as a fatigue crack, nonlinear ultrasonic techniques, which look for nonlinear characteristics of ultrasounds, have gained prominence in structural health monitoring (SHM) and nondestructive testing (NDT) applications [1,2,3,4]. Nonlinear ultrasonic techniques use nonlinear components, such as harmonics and modulations, which are generated from the interaction of ultrasounds with nonlinear sources such as material degradation and fatigue cracks.

One of the essential steps for using guided wave based nonlinear ultrasonic techniques on plate, rod, and pipe structures is matching the necessary conditions (NCs) for the generation of the nonlinear components. The NCs, which must be satisfied for the creation of nonlinear components due to distributed material nonlinearity are provided by de Lima and Hamilton [5,6]. The references show that, when the NCs are met, the amplitude of the nonlinear component increases in proportion to the wave propagation distance (i.e., cumulative characteristics). Additionally, Srivastava and di Scalea theoretically and experimentally show that the even order nonlinear harmonics exist only for symmetric (S) Lamb wave modes, but not for anti-symmetric (A) modes in plate-like structures [7]. There is a large volume of literature utilizing the NCs for the distributed material nonlinearity to characterize material properties and degradations [8,9,10,11,12,13].

For a localized nonlinear source such as a fatigue crack, Zaitsev et al. show that the crack surfaces should be oscillated by the applied ultrasound inputs for the generation of nonlinear modulation components [14]. Furthermore, Klepka et al. experimentally demonstrate that the nonlinear modulation amplitude strongly depends on the types of crack motions produced by applied vibrations [15]. However, the NCs for localized crack nonlinearity have not been explicitly formulated nor fully validated considering both propagating waves and stationary vibrations so far.

In this paper, the NCs for the generation of nonlinear ultrasonic modulation are formulated specifically for a localized nonlinear source in a plate-like structure. Then, the suitability of the NCs is experimentally validated using ultrasounds obtained from an aluminum plate with a real fatigue crack. The uniqueness of this paper lies in (1) formulation of the NCs for the nonlinear ultrasonic generation at the presence of a localized fatigue crack in a plate-like structure; (2) consideration of both transient propagating waves and stationary vibrations; (3) comparison of the effects of distributed and localized nonlinear sources on the NCs; and (4) experimental validation of the NCs using aluminum plate specimens focusing on nonlinear modulation.

This paper is organized as follows. In Section 2, the NCs for distributed material nonlinearity are reviewed, and the NCs for localized crack nonlinearity are formulated. Section 3 and Section 4 describe the experimental setup and test results performed for validating the proposed NCs, respectively. The conclusion and discussions are provided in Section 5.

## 2. Theoretical Development

In this section, first, the NCs for distributed material nonlinearity are reviewed with relevant references. Then, based on the assumption that input ultrasounds fluctuate the elastic modulus at the crack location due to contact or friction, the NCs for a localized crack nonlinearity are developed.

### 2.1. Working Principle of Nonlinear Ultrasound

When two waves a and b at distinct frequencies $\omega_{a}$ and $\omega_{b}$ ($\omega_{a}<\omega_{b}$) are applied to a plate-like structure without any nonlinear source and propagated in the x-direction, the displacement induced by the input waves can be expressed as

(1) $$u_{0}=A_{0}\exp\left(i\left(\kappa_{a}x-\omega_{a}t\right)\right)+B_{0}\exp\left(i\left(\kappa_{b}x-\omega_{b}t\right)\right)$$

where $A_{0}$ and $B_{0}$ are the amplitudes, $\kappa_{a}$ and $\kappa_{b}$ are the wavenumbers of the waves a and b, respectively.

When the waves are applied to the structure with either a distributed or a localized nonlinear source and the corresponding NCs are matched, the displacement of waves after passing the nonlinear source, $u_{1}$, can be represented as the summation of the linear, $u_{1}^{L}$, harmonics, $u_{1}^{H}$, and modulation, $u_{1}^{M}$, components due to the interaction of the input waves with the nonlinear source [5,14,15,16]

(2) $$u_{1}=u_{1}^{L}+u_{1}^{H}+u_{1}^{M}$$

where

(3) $$u_{1}^{L}=A_{1}^{L}\exp\left(i\left(\kappa_{a}x-\omega_{a}t\right)\right)+B_{1}^{L}\exp\left(i\left(\kappa_{b}x-\omega_{b}t\right)\right)$$

(4) $$u_{1}^{H}=A_{1}^{H}\exp\left(2i\left(\kappa_{a}x-\omega_{a}t\right)\right)+B_{1}^{H}\exp\left(2i\left(\kappa_{b}x-\omega_{b}t\right)\right)$$

and

(5) $$u_{1}^{M}=A_{1}^{M}\exp\left(i\left[\left(\kappa_{b}\pm \kappa_{a}\right)x-\left(\omega_{b}\pm \omega_{a}\right)t\right]\right)$$

where $A_{1}^{L}$ and $A_{1}^{H}$ are the amplitudes of the linear component at $\omega_{a}$ and the second harmonics at $2\omega_{a}$ due to the nonlinear source, respectively. $B_{1}^{L}$ and $B_{1}^{H}$ are defined similarly. $A_{1}^{M}$ is the amplitude of the first spectral sidebands (modulation) at $\omega_{b}\pm \omega_{a}$ due to the mutual interaction of the input waves at the nonlinear source. In this paper, higher order harmonic and modulation components are omitted for simplicity. For the experimental validation of the proposed NCs, only the $A_{1}^{M}$ component is extracted and used.

When two waves at the same frequency propagating in opposite directions are superimposed by reflections at structural boundaries, the waves create standing waves, eventually converging to vibration modes [17]. For example, the modulation component becomes

(6) $$u_{1}^{M}={\bar{A}}_{1}^{M}\cos\left(\left(\kappa_{b}\pm \kappa_{a}\right)x\right)\exp\left(-i\left(\omega_{b}\pm \omega_{a}\right)t\right)$$

where ${\bar{A}}_{1}^{M}$ is the vibration amplitude at $\omega_{b}\pm \omega_{a}$, which can be interpreted as the amplitude of the frequency response function (FRF) at $\omega_{b}\pm \omega_{a}$, and $\cos\left(\left(\kappa_{b}\pm \kappa_{a}\right)x\right)$ is the corresponding vibration mode shape of the structure at $\omega_{b}\pm \omega_{a}$.

It is known that both distributed material nonlinearity and a localized crack can produce the nonlinear components, and the relevant findings can be summarized as follows:

1. Distributed material nonlinearity: A crystallographic defect, or irregularity within a material such as dislocation or interatomic potential distributed over the entire material can be a source of nonlinearity. In addition, distributed initial micro cracks/voids in the material also contribute to nonlinearity. The distributed material nonlinearity is known to be weak and not localized (global characteristic), in comparison to the nonlinearity caused by localized damage such as a fatigue crack [18,19]. However, this nonlinearity can occasionally make a significant contribution to the measured nonlinear components [14].
2. Localized crack nonlinearity: When ultrasonic waves or vibrations are applied to a structure with a crack, they cause the crack surface to alternate between open and closed (contact) conditions. This is called a ‘breathing crack’ or ‘contact acoustic nonlinearity’ (CAN) [14,15,16,20]. The contacts between rough crack surfaces, called ‘micro-contact’, can also act as a localized nonlinear source even when the crack is not completely open and closed [21]. Additionally, it is demonstrated that dissipative mechanisms (friction) between the crack surfaces can cause nonlinearity [15]. The nonlinearity due to the crack opening/closing or the friction are known to be localized and stronger than the distributed material nonlinearity [15,19].

### 2.2. NCs for Nonlinear Ultrasonic Modulation Given Distributed Material Nonlinearity

The NCs for the distributed material nonlinearity in a plate-like structure have been theoretically and experimentally investigated by several researchers, and the findings can be summarized as below:

1. Synchronism condition: In the propagating waves, the phase velocities of low frequency (LF) and high frequency (HF) inputs, $\omega_{a}$ and $\omega_{b}$, should be identical to the phase velocity at $\omega_{b}\pm \omega_{a}$ [5,6]. From the viewpoint of vibration, the point-wise multiplication of the vibration mode shapes of LF and HF input signals should become the mode shape of the vibration at $\omega_{b}\pm \omega_{a}$ [22].
2. Non-zero power flux condition: From the wave propagation point of view, the mode type of two input waves should match with the mode type of the modulation wave so that the energy from the input waves can be readily transmitted to the nonlinear modulation wave [5,6]. In a plate-like structure, nonlinear harmonics exist only for S Lamb wave modes, but not for A Lamb wave modes at even order harmonics (2ω, 4ω, …) [7]. Similarly, the first modulation component ($\omega_{b}\pm \omega_{a}$) will not be generated when both LF and HF inputs are A Lamb wave modes. For vibrations, the mode type (longitudinal or flexural) should also match in the longitudinal direction in addition to the thickness direction.

### 2.3. NCs for Nonlinear Ultrasonic Modulation Given Localized Crack Nonlinearity

In this subsection, the NCs for the generation of ultrasonic modulation are formulated assuming the presence of a localized nonlinear source. The stress induced by the input waves propagating in the x-direction without any nonlinear source can be written as

(7) $$\sigma_{0}=E_{0}\frac{\partial u_{0}}{\partial x}=E_{0}\left[A_{0}^{L}\exp\left(i\left(\kappa_{a}x-\omega_{a}t\right)\right)+B_{0}^{L}\exp\left(i\left(\kappa_{b}x-\omega_{b}t\right)\right)\right]i$$

where $E_{0}$ is the elastic modulus of the structure. When a localized nonlinear source such as a fatigue crack is introduced at $x_{0}$ and oriented perpendicular to the wave propagation direction (the z-direction), the average elastic modulus at $x_{0}$ is reduced from $E_{0}$ to $\bar{E}$ and the instantaneous elastic modulus, $E_{1}\left(x_{0}\right)$, fluctuates around $\bar{E}$ in proportion to $u_{0}$ due to CAN (crack mode I) [16]:

(8) $$\;E_{1}\left(x_{0}\right)=\bar{E}\left(x_{0}\right)+\alpha E_{0}\frac{\partial u_{0}\left(x_{0}\right)}{\partial x}=\;\left[1-\alpha \max\left(\frac{\partial u_{0}\left(x_{0}\right)}{\partial x}\right)\right]E_{0}+\alpha E_{0}\frac{\partial u_{0}\left(x_{0}\right)}{\partial x}$$

where $\bar{E}\left(x_{0}\right)$ is the average elastic modulus after the fatigue crack formation at $x_{0}$ and α is the nonlinear coefficient for representing the nonlinearity due to the fatigue crack, respectively. The max operation finds the maximum strain induced by the input waves at the crack location. Then, the stress induced by the input waves at the crack location, $\sigma_{1}\left(x_{0}\right)$, can be expressed as

(9) $$\sigma_{1}\left(x_{0}\right)=E_{1}\left(x_{0}\right)\frac{\partial u_{0}\left(x_{0}\right)}{\partial x}=\left[1-\alpha \max\left(\frac{\partial u_{0}\left(x_{0}\right)}{\partial x}\right)\right]E_{0}\frac{\partial u_{0}\left(x_{0}\right)}{\partial x}+\;\alpha E_{0}{\left(\frac{\partial u_{0}\left(x_{0}\right)}{\partial x}\right)}^{2}$$

Here, the second term on the right hand side of Equation (9) represents the nonlinear component due to the crack and it should be non-zero for the generation of nonlinear modulation at the crack location. Thus, for the generation of nonlinear modulation

(10) $$\frac{\partial u_{0}\left(x_{0}\right)}{\partial x}\neq 0$$

The NCs for localized crack nonlinearity in a plate-like structure can be proposed as follows:

1. Crack perturbation condition: The strain at the crack location should be oscillated by both input ultrasounds. In stationary vibration, the node is a point where the wave has the minimum (zero) amplitude. Thus, the nodal positions correspond to zero strain, whereas anti-nodes correspond to the maximum strain. This condition indicates that the nonlinear modulation is not generated when the crack is located at one of the nodes of the input vibration modes, where $\partial u_{0}\left(x_{0}\right)/\partial x=0$. As for transient wave propagation, this condition is unconditionally satisfied because the strain at the crack is always perturbed by the propagating input waves.
2. Mode matching condition: The crack motion induced by one of the input ultrasounds should modulate the other ultrasound at the crack location. For example, assume that an LF shear horizontal (i.e., $\partial u_{0}\left(x_{0}\right)/\partial x=0$, $\partial u_{0}\left(x_{0}\right)/\partial y\neq 0$) and a HF longitudinal (i.e.,$\;\partial u_{0}\left(x_{0}\right)/\partial x\neq 0$, $\partial u_{0}\left(x_{0}\right)/\partial y=0$) waves are propagated in the x-direction through a localized crack whose orientation is perpendicular to the wave propagating direction (the z-direction). Then, the LF shear horizontal wave causes the crack surface to oscillate in the y-direction (friction, crack mode II). However, this crack motion does not modulate the HF longitudinal wave nor create the modulation component, because their movements are orthogonal to each other. A previous investigation also demonstrates that the high amplitude of the nonlinear ultrasonic modulation is observed when the ultrasonic signal is modulated due to the crack motion [15].

## 3. Experimental Setup

### 3.1. Description of Experimental Setup and Specimen

**Figure 1:**
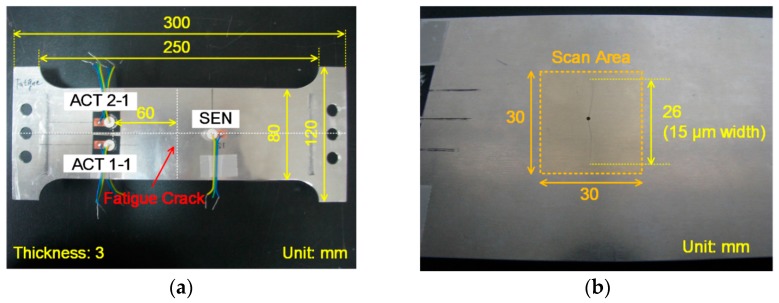
Aluminum plate specimen. (**a**) The geometry and dimensions; (**b**) A close-up of the fatigue crack.

An aluminum (6061-T6) plate specimen with 3 mm thickness was fabricated as shown in Figure 1a. 80,000 cycles of 4–40 kN (R = 0.1) tensile loadings with a 10 Hz cycle rate were applied to the specimen for introducing a fatigue crack. The fatigue crack initiated from the hole at the center of the specimen and grew up to 26 mm long and 15 μm wide as shown in Figure 1b. The crack length and width were measured using optical microscopic images. Five identical APC850 piezoelectric transducers (PZTs) (∅ = 10 mm, t = 0.5 mm) manufactured by APC International were installed on the specimen. A pair of PZTs labeled as ACT 1-1 and 1-2 were collocated but placed on the opposite sides of the specimen for LF excitation of selective S and A Lamb wave modes [23]. Similarly, a pair of ACT 2-1 and 2-2 were installed for the selective HF Lamb wave mode generation. The corresponding responses were obtained at SEN with the distance of 9 cm from the excitation PZTs. For the data acquisition, a NI PXI system consisting of two arbitrary waveform generators and a high speed digitizer was used [24]. LF and HF inputs had peak-to-peak voltages of 80 V and 60 V, respectively. Based on the material properties of the PZTs provided by the manufacturer, the theoretical maximum displacement of the specimen induced by the PZT excitation was around 0.3 μm, and this level of displacement was large enough to generate both the crack nonlinearity and the material nonlinearity as provided in [15]. Furthermore, the displacement was magnified when a continuous input (vibration) was applied at one of the resonance frequencies of the structure. The inputs were converted to analog signals with a 2 MHz conversion rate, and the responses were measured simultaneously at a 2 MHz sampling rate as an average of 10 repetitions.

For the generation of stationary vibrations, a sine signal with 0.5 s duration was applied to ensure steady-state vibration responses of the specimen. For the wave generation, a tone-burst signal with 0.1 ms duration was applied. The duration of the tone-burst input was determined so that the reflections from the boundaries of the specimen did not overlap with the first arrival wave packet. The obtained ultrasounds were analyzed in the frequency domain by applying a fast Fourier transform (FFT) for the vibration and a short time Fourier transform (STFT) for the wave propagation up to the first arrival wave packet. For FFT and STFT, the maximum integer number of cycles were used to avoid leakage in the spectral analysis and the frequency resolution was 2 Hz. The responses were normalized with respect to the product of the HF and LF input amplitudes to minimize the variation of modulation amplitude under changing input frequencies.

### 3.2. Determination of Input Frequencies

**Figure 2:**
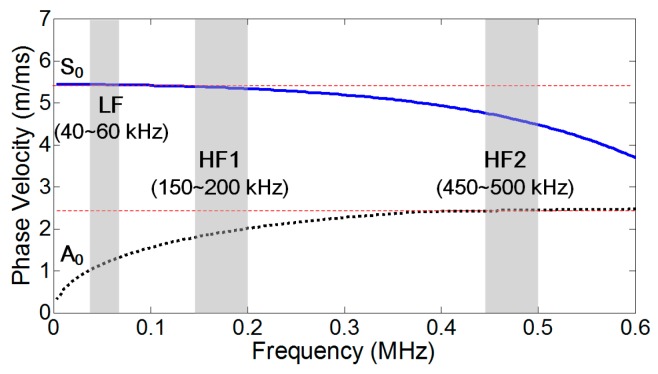
Phase velocity dispersion curve of the specimen: LF input was selected between 40–60 kHz, and HF input between 150–200 kHz and 450–500 kHz.

Before the validation of the NCs for nonlinear modulation component generation, dispersion curves for phase velocities were experimentally obtained from the specimen as shown in Figure 2. Here, the dispersion curves for S and A Lamb wave modes were obtained by selectively exciting the S and A modes using the collocated PZTs on the opposite sides of the specimen as described in the previous section. For example, the frequency bands for LF (40–60 kHz) and HF1 (150–200 kHz) were selected so that the S_0_ modes in HF1 ranges satisfied the synchronism condition [25]. The low dispersion region at HF1 did not affect matching the synchronism condition much because the wave propagation distance in this study was relatively short (80 mm). Additionally, the S_0_ modes in HF2 (450–500 kHz) were selected to avoid the synchronism condition. Once the LF, HF1, and HF2 ranges are determined, actual specific input frequency values were determined by selecting resonance frequencies within each frequency band through experimental modal tests.

For the validation of the crack perturbation condition, the experimental modal analysis was conducted using a 3D Laser Doppler Vibrometer (LDV, PSV400, Polytec GmbH, Waldbronn, Germany). With scanning capability, LDV can visualize propagating ultrasonic waves and vibration modes with high spatial resolution [26]. When a laser beam is reflected from a vibrating target surface, the frequency of the returned laser beam is shifted based on the Doppler Effect. Using three co-aligned laser beams, 3D LDV can measure not only out-of-plane (the z-direction) dominant A_0_ mode but also in-plane (the x or y-direction) dominant S_0_ mode motions [27].

The 3D LDV was installed 0.9 m apart from the specimen, and the ultrasound responses were measured with a 2.56 MHz sampling rate. A VD-09 20 mm/s/V internal decoder, which has a maximum sensitivity of 20 mm/s/V up to 1 MHz, was used for the measurement. A 12.8 ms linear chirp signal with the frequency ranges selected from the dispersion curves in Figure 2 was applied through the PZTs on the specimen. As shown in Figure 1b, 30 × 30 mm^2^ square area near the center hole of the specimen was scanned with 1.5 mm spatial resolution (20 × 20 scan points). To improve the signal-to-noise ratio, the responses at each scanning point were measured 200 times and averaged in the time domain.

**Figure 3:**
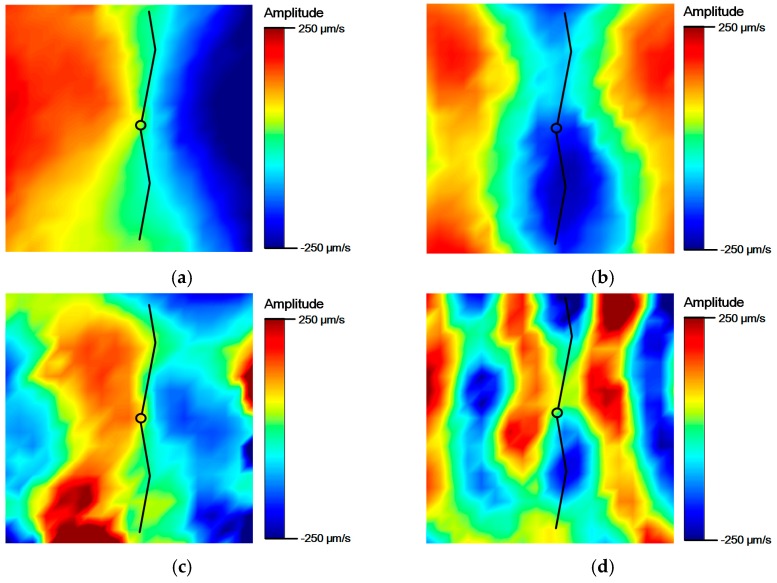
Vibration modes of the aluminum specimen using a 3D Laser Doppler Vibrometer (LDV) scanning. (**a**) 46 kHz S_0_ mode (node at the crack); (**b**) 50 kHz A_0_ mode (anti-node at the crack); (**c**) 170 kHz S_0_ mode (node at the crack); (**d**) 181 kHz A_0_ mode (anti-node at the crack).

Figure 3 shows the results of the experimental modal analysis. For the validation of the crack perturbation condition, the input frequencies were selected so that the crack is located either at a node or at an anti-node of the induced vibration modes. For example, when LF input is set to 46 kHz, which corresponds to one of the resonance frequencies of S_0_ mode, the crack is located at the node of the vibration mode as shown in Figure 3a. On the other hand, when HF input is tuned to 181 kHz coinciding with one of the resonance frequencies of A_0_ mode, the crack is placed at the anti-node as shown in Figure 3d. Other input frequencies for all the cases listed in Table 1 were determined in a similar manner.

## 4. Experimental Results

### 4.1. Crack Perturbation Condition

**Figure 4:**
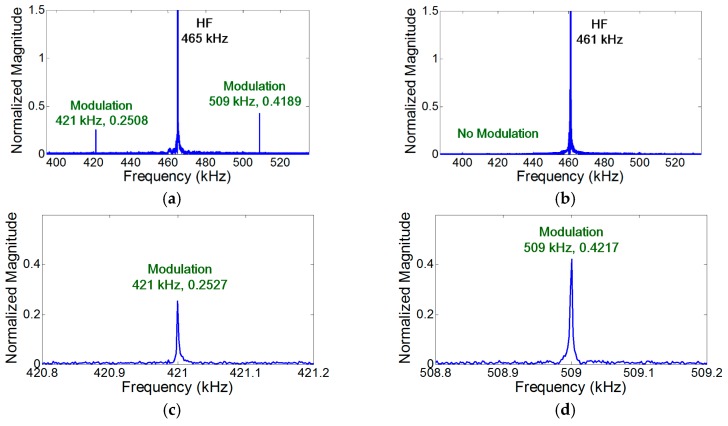
Experimental validation of the crack perturbation condition. The modulation occurs only when the crack is located at the anti-nodes of both LF and HF vibration modes. Here, S_0_ modes are excited at both LF and HF inputs, and the synchronism condition is avoided to minimize the effect of distributed material nonlinearity. (**a**) Crack is located at the anti-nodes of both LF (44 kHz) and HF (465 kHz) vibration modes; (**b**) Crack is located at the nodes of both LF (46 kHz) and HF (461 kHz) vibration modes; (**c**) Close-up of (a) near 421 kHz; (**d**) Close-up of (a) near 509 kHz.

**Figure 5:**
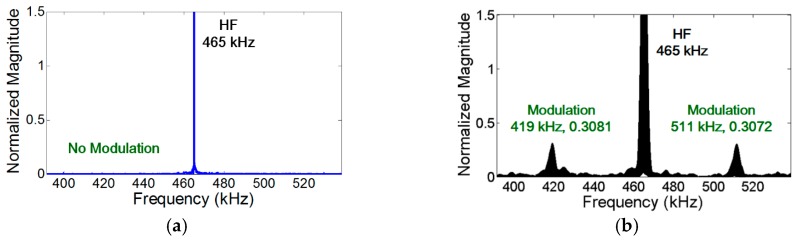
Experimental comparison of the effects of stationary vibration and propagating wave on the nonlinear modulation by converting LF input from a stationary sinusoidal input to a transient wave form. The modulation does not occur when the crack is located at the node of LF vibration mode. However, when the sinusoidal LF input is converted to a transient wave, the modulation appears. Here, both 46 kHz LF and 465 kHz HF modes are S_0_ modes, and the synchronism condition is avoided to minimize the effect of distributed material nonlinearity. (**a**) Crack is located at the node of LF vibration mode and the anti-node of HF mode; (**b**) LF input is converted from a stationary sinusoidal input in (a) to a transient wave.

**Figure 6:**
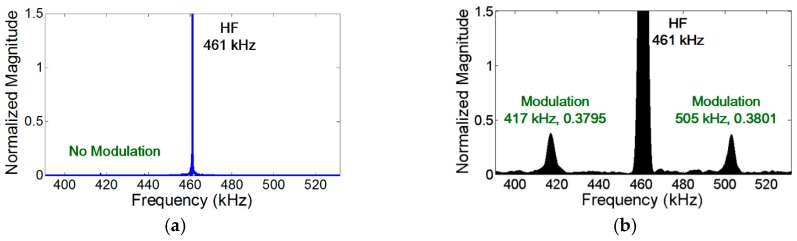
Experimental comparison of the effects of propagating wave and stationary vibration on modulation by converting HF input from a stationary sinusoidal input to a transient wave form. The modulation does not occur when the crack is located at the node of HF vibration mode. However, when the sinusoidal HF input is converted to a transient wave form, the modulation appears. Here, both 44 kHz LF and 461 kHz HF modes are S_0_ modes, and the synchronism condition is avoided to minimize the effect of distributed material nonlinearity. (**a**) Crack is located at the anti-node of LF vibration mode and the node of HF mode; (**b**) HF input is converted from a stationary sinusoidal input in (a) to a transient wave.

To validate the crack perturbation condition, two sinusoidal vibration inputs are applied to the specimen and the generation of the modulation components is examined. Here, LF and HF input frequencies are selected and symmetrically excited by the collocated PZTs on both sides of the specimen so that only S_0_ modes were generated at both input frequencies. In addition, the synchronism condition is avoided to minimize the effect of distributed material nonlinearity for all the subsequent experiments unless explicitly stated differently. As shown in Figure 4a, the modulation occurs when the crack is located at the anti-nodes of both LF and HF vibration modes. However—as shown in Figure 4b, Figure 5a, and Figure 6a—the modulation does not occur where the crack is located at least at one of the nodes of LF and HF vibration modes. When the crack is located at one of the vibrational nodes, the crack is not oscillated by one of the input vibration modes, no crack motion occurs, and the other vibration mode is not modulated by the crack.

Figure 4a–d validate that the length of the time signal, the sampling rate, and the frequency resolution for the spectral analysis are properly selected to capture the presence of modulation components.

**Figure 7:**
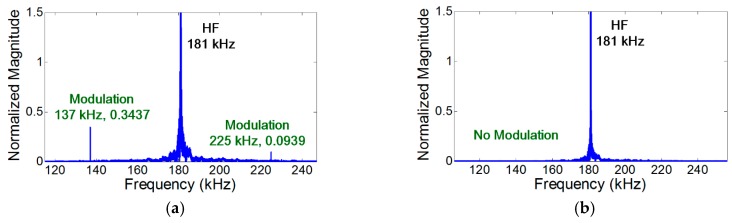
Experimental validation of the mode matching condition. The modulation does not occur when the modes of both LF and HF are A_0_. Here, the crack is located at the anti-nodes of both LF and HF vibration modes, and the synchronism condition is avoided to minimize the effect of distributed material nonlinearity. (**a**) LF (44 kHz) and HF (181 kHz) inputs generate S_0_ and A_0_ modes, respectively; (**b**) Both LF (50 kHz) and HF (181 kHz) inputs generate A_0_ modes.

**Figure 8:**
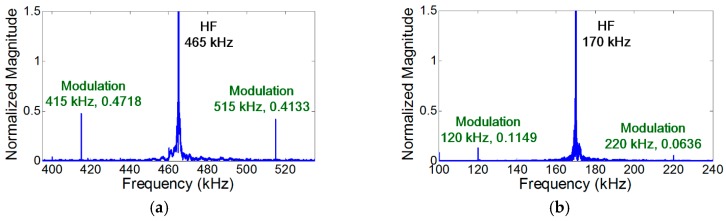
Experimental comparison of the NCs for distributed material and localized crack nonlinearities. Here, A_0_ mode LF and S_0_ mode HF are applied, respectively. (**a**) The anti-nodes of both LF (50 kHz) and HF (465 kHz) vibration modes when the NCs for localized crack nonlinearity are matched and the NCs for distributed nonlinearity are avoided; (**b**) The anti-nodes of LF (50 kHz) vibration mode and the node HF (170 kHz) modes when the NCs for distributed material nonlinearity are matched and the NCs for localized crack are avoided.

In the case of vibration, the amplitude of the modulation component is further amplified when the modulation frequency coincides with one of the resonance frequencies of the structure (i.e., nonlinear resonance) [22]. Figure 4a, Figure 7a, and Figure 8a show that the amplitudes of two modulation components at $\omega_{b}\pm \omega_{a}$ are different, because their amplitudes change depending on the relative positions of their modulation frequencies with respect to the resonance frequencies of the structures. On the other hand, it is observed in Figure 5b and Figure 6b that the amplitudes of the two modulation frequencies are practically identical for propagating waves.

### 4.2. Effects of Propagating Wave vs. Stationary Vibration on Nonlinear Ultrasonic Modulation

Next, the effects of stationary vibration and propagating wave on the crack perturbation condition are investigated by converting a stationary sinusoidal input signal into a transient propagating wave form. Here, both LF and HF inputs were S_0_ modes. As shown in Figure 5a, the modulation does not occur when the crack is located at the node of LF vibration mode and the anti-node of HF mode. However, when the LF input is converted to a transient wave form, the modulation appears as shown in Figure 5b. Similarly, the modulation does not occur when the crack is located at the node of HF vibration mode and the anti-node of LF mode as shown in Figure 6a. When the HF input is converted to a transient wave form, the modulation appears as presented in Figure 6b. The modulation also occurs when both inputs are transient waves. This particular case corresponds to Case 18 of Table A1 in the Appendix. All the results substantiate that the crack perturbation condition is always satisfied when both input signals are propagating waves instead of stationary vibrations.

### 4.3. Mode Matching Condition

The mode matching condition is experimentally validated using the cases where the crack is located at the anti-nodes of both LF and HF vibration modes. The experimental results shown in Figure 7 indicate that the modulation does not occur when the mode of both LF and HF inputs are A_0_. Additional tests were conducted to validate the mode matching condition and summarized in the Appendix (Cases 25–40). The experimental results substantiate the fact that, for the generation of nonlinear modulation at a localized fatigue crack, one of the input ultrasounds should produce the crack motion that can subsequently modulate the other input ultrasound.

### 4.4. Effects of Distributed vs. Localized Nonlinear Sources on Nonlinear Ultrasonic Modulation

Finally, the generation of nonlinear modulation due to distributed material and localized nonlinear sources are compared. Figure 8 shows the experimental results when the modes of LF and HF inputs are A_0_ and S_0_, respectively. In Figure 8a, the modulation occurs when the crack is located at the anti-nodes of both LF and HF vibration modes. Here, the NCs for localized crack nonlinearity are matched and the NCs for distributed nonlinearity are avoided.

It has been reported that the modulation produced by intrinsic distributed material nonlinearity is much smaller than that produced by a localized nonlinear source such as a fatigue crack [15,16,18,19,20]. However, Figure 8b shows that the modulation produced by the material nonlinearity is still visible when the synchronism condition is matched and the NCs for localized cracks are avoided. Furthermore, Figure 5a, Figure 6a, and Figure 7b show that the modulation does not occur when the NCs for distributed material nonlinearity and localized cracks are avoided.

**Figure 9:**
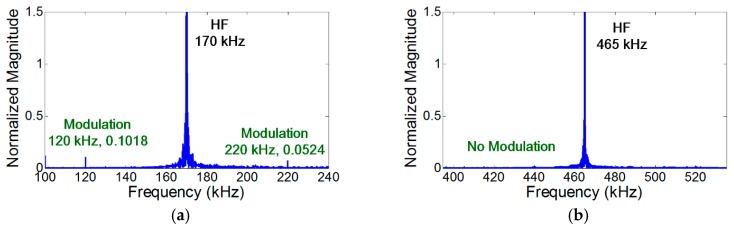
Experimental validation of the presence of the distributed material nonlinearity using the aluminum specimen without a fatigue crack. The modulation does not occur when the NCs for distributed material nonlinearity are avoided. However, when the NCs for that are matched, the modulation components are generated without any fatigue crack. (**a**) NCs for the distributed material nonlinearity are matched (same with Figure 8b, without fatigue crack); (**b**) NCs for the distributed material nonlinearity are avoided (same with Figure 4a, without fatigue crack).

Note that the presence of the distributed material nonlinearity was experimentally validated using the identical aluminum plate specimen without a fatigue crack. All the test setups were identical to Figure 4a and Figure 8b. Figure 9a shows that the modulation, whose level is equivalent to that of Figure 8b, appears when the NCs for distributed material nonlinearity are matched. On the other hand, the modulation does not occur in Figure 9b when the NCs for distributed material nonlinearity are avoided (even the input frequencies and modes are identical to Figure 4a). Thus, for reliable crack detection using nonlinear modulation, the NCs for distributed material nonlinearity such as synchronism and non-zero power flux conditions should be avoided.

## 5. Conclusions

In this study, the conditions of nonlinear ultrasonic modulation generation in a plate-like structure with a localized nonlinear source—such as a fatigue crack—are investigated. First, the necessary condition (NCs), which are necessary for the generation of nonlinear ultrasonic components are formulated for a localized nonlinear source. Then, the suitability of the NCs is validated through the experiments obtained from aluminum plate specimens with a real fatigue crack specially for nonlinear ultrasonic modulation. Based on the theoretical formulation and the experimental validation, the following guidelines for effective crack detection are provided. First, the NCs for distributed material nonlinearity such as synchronism and non-zero power flux conditions should be avoided for reliable crack detection. Second, the employment of transient wave inputs rather than stationary vibration inputs can be more effective when the location of the crack is unknown. However, the vibration excitation can exert a large energy into a target structure and it is easier to perturb the crack when the crack is located at the anti-node of the resonance vibration modes of the structure. Investigation of various LF and HF frequency combinations can minimize the node/anti-node effect and provide more reliable crack detection result for real application.

The uniqueness of this paper lies in (1) formulation of the NCs for the nonlinear ultrasonic components generation at the presence of a localized fatigue crack in a plate-like structure; (2) comparison of the NCs when transient waves and stationary vibration inputs are applied; (3) comparison of the NCs when the source of nonlinearity is either distributed or localized; and (4) experimental validation of the NCs using aluminum plate specimens, especially for nonlinear ultrasonic modulation.

## Figures and Tables

**Table 1:** Input frequencies determined by experimental modal analysis.

Case #	Input	Frequency	Mode	Motion at Crack
**1**	LF	44 kHz	S_0_	Anti-node
**2**	LF	46 kHz	S_0_	Node
**3**	LF	50 kHz	A_0_	Anti-node
**4**	LF	53 kHz	A_0_	Node
**5**	HF1	169 kHz	S_0_	Anti-node
**6**	HF1	170 kHz	S_0_	Node
**7**	HF2	465 kHz	S_0_	Anti-node
**8**	HF2	461 kHz	S_0_	Node
**9**	HF1	181 kHz	A_0_	Anti-node
**10**	HF1	183 kHz	A_0_	Node
**11**	HF2	482 kHz	A_0_	Anti-node
**12**	HF2	485 kHz	A_0_	Node

## Appendix A

**Table A1:** Nonlinear ultrasonic modulation generation experimental results.

Case#	LF			HF			Sync. Condition Match	Modulation (Source)
	Freq. (kHz)	Mode	Crack Motion	Freq. (kHz)	Mode	Crack Motion		
**1**	44	S_0_	Anti-node	169	S_0_	Anti-node	O	O (both)
**2**	44	S_0_	Anti-node	465	S_0_	Anti-node	X	O (crack)
**3**	46	S_0_	Node	169	S_0_	Anti-node	O	O (material)
**4**	46	S_0_	Node	465	S_0_	Anti-node	X	X
**5**	44	S_0_	Anti-node	170	S_0_	Node	O	O (material)
**6**	44	S_0_	Anti-node	461	S_0_	Node	X	X
**7**	46	S_0_	Node	170	S_0_	Node	O	O (material)
**8**	46	S_0_	Node	461	S_0_	Node	X	X
**9**	44	S_0_	Anti-node	170	S_0_	- (Wave)	-	O (crack)
**10**	44	S_0_	Anti-node	461	S_0_	- (Wave)	-	O (crack)
**11**	46	S_0_	Node	170	S_0_	- (Wave)	-	X
**12**	46	S_0_	Node	461	S_0_	- (Wave)	-	X
**13**	46	S_0_	- (Wave)	169	S_0_	Anti-node	-	O (crack)
**14**	46	S_0_	- (Wave)	465	S_0_	Anti-node	-	O (crack)
**15**	46	S_0_	- (Wave)	170	S_0_	Node	-	X
**16**	46	S_0_	- (Wave)	461	S_0_	Node	-	X
**17**	46	S_0_	- (Wave)	170	S_0_	- (Wave)	O	O (both)
**18**	46	S_0_	- (Wave)	461	S_0_	- (Wave)	X	O (crack)
**19**	44	S_0_	Anti-node	482	A_0_	Anti-node	O	O (both)
**20**	44	S_0_	Anti-node	181	A_0_	Anti-node	X	O (crack)
**21**	50	A_0_	Anti-node	169	S_0_	Anti-node	O	O (both)
**22**	50	A_0_	Anti-node	465	S_0_	Anti-node	X	O (crack)
**23**	50	A_0_	Anti-node	482	A_0_	Anti-node	O	X
**24**	50	A_0_	Anti-node	181	A_0_	Anti-node	X	X
**25**	44	S_0_	Anti-node	485	A_0_	- (Wave)	-	O (crack)
**26**	44	S_0_	Anti-node	183	A_0_	- (Wave)	-	O (crack)
**27**	50	A_0_	Anti-node	170	S_0_	- (Wave)	-	O (crack)
**28**	50	A_0_	Anti-node	461	S_0_	- (Wave)	-	O (crack)
**29**	50	A_0_	Anti-node	485	A_0_	- (Wave)	-	X
**30**	50	A_0_	Anti-node	183	A_0_	- (Wave)	-	X
**31**	53	A_0_	- (Wave)	169	S_0_	Anti-node	-	O (crack)
**32**	53	A_0_	- (Wave)	465	S_0_	Anti-node	-	O (crack)
**33**	53	A_0_	- (Wave)	482	A_0_	Anti-node	-	X
**34**	53	A_0_	- (Wave)	181	A_0_	Anti-node	-	X
**35**	46	S_0_	- (Wave)	485	A_0_	- (Wave)	X	X
**36**	46	S_0_	- (Wave)	183	A_0_	- (Wave)	X	X
**37**	53	A_0_	- (Wave)	170	S_0_	- (Wave)	X	X
**38**	53	A_0_	- (Wave)	461	S_0_	- (Wave)	X	X
**39**	53	A_0_	- (Wave)	485	A_0_	- (Wave)	X	X
**40**	53	A_0_	- (Wave)	183	A_0_	- (Wave)	X	X
**41**	46	S_0_	Node	485	A_0_	Node	O	O (material)
**42**	44	S_0_	Anti-node	485	A_0_	Node	O	O (material)
**43**	46	S_0_	Node	482	A_0_	Anti-node	O	O (material)
**44**	53	A_0_	Node	170	S_0_	Node	O	O (material)
**45**	50	A_0_	Anti-node	170	S_0_	Node	O	O (material)
**46**	53	A_0_	Node	169	S_0_	Anti-node	O	O (material)
**47**	53	A_0_	Node	485	A_0_	Node	O	X
**48**	50	A_0_	Anti-node	485	A_0_	Node	O	X
**49**	53	A_0_	Node	482	A_0_	Anti-node	O	X
